# Retraction: MicroRNA-503 Acts as a Tumor Suppressor in Osteosarcoma by Targeting L1CAM

**DOI:** 10.1371/journal.pone.0269900

**Published:** 2022-06-08

**Authors:** The PLOS ONE Editors

Following the publication of this article [1], similarities were noted between this article and articles submitted by other research groups, including [2–10], of which one article [10] was previously retracted [11].

Similarities included the following figures, which appear to fully or partially overlap, despite being published in different articles and representing different conditions:

- untreat panel in Fig 3A of [1], and pCDNA-MDM2 + scramble panel in Fig 4C of [2].
- untreat and control panels in Fig 3B of [1], and pCDNA-MDM2 + miR-410 panel in Fig 4C of [2].
- inhibitors panel in Fig 3B of [1], and miR-575 mimic panels in Fig 2C and 2D of [8].
- L1CAM panel in Fig 4D of [1], WASF3 panel in Fig 3D of [4], PAQR3 panel in Fig 4D of [6], and BRD7 panel in Fig 4C of [7].

The corresponding author did not comment on the above concerns. They provided data files which did not appear to contain the full raw data underlying the published figure, and they stated that they are unable to provide the ethics approval documentation. Therefore, the concerns regarding the similarities between this article [1] and others [2–10] remain unresolved.

The unresolved concerns call into question the validity and provenance of the reported results, and the adherence of this article to the PLOS Authorship policy. Therefore, the *PLOS ONE* Editors retract this article [1].

All authors did not comment on the retraction decision, did not respond directly or could not be reached.
